# A flexible readout mechanism of human sensory representations

**DOI:** 10.1038/s41467-019-11448-7

**Published:** 2019-08-02

**Authors:** Daniel Birman, Justin L. Gardner

**Affiliations:** Department of Psychology, Stanford University, Stanford, CA 94305 USA

**Keywords:** Attention, Computational neuroscience, Visual system, Perception

## Abstract

Attention can both enhance and suppress cortical sensory representations. However, changing sensory representations can also be detrimental to behavior. Behavioral consequences can be avoided by flexibly changing sensory readout, while leaving the representations unchanged. Here, we asked human observers to attend to and report about either one of two features which control the visibility of motion while making concurrent measurements of cortical activity with BOLD imaging (fMRI). We extend a well-established linking model to account for the relationship between these measurements and find that changes in sensory representation during directed attention are insufficient to explain perceptual reports. Adding a flexible downstream readout is necessary to best explain our data. Such a model implies that observers should be able to recover information about ignored features, a prediction which we confirm behaviorally. Thus, flexible readout is a critical component of the cortical implementation of human adaptive behavior.

## Abstract (web summary)

It is known that attention can modify the brain's representations of sensory stimuli to enhance features of importance. Here, the authors show that flexible readout of cortical representations is also required to explain the behavioral effects of attention.

## Introduction

Humans can flexibly attend to different aspects of the environment when their goals require it. This can be operationalized by asking human observers to report about one feature of a visual stimulus while ignoring other features. Such context-dependent judgments could be supported by a cortical implementation which increases sensitivity or selectivity for the sensory representations of reported features while suppressing others. A second and potentially complimentary implementation is to maintain stable sensory representations while flexibly changing the downstream readout of these.

A great deal of evidence exists for the former possibility of changing representations to accommodate behavioral demands. Behavioral manipulations of spatial attention^1–4^, feature-based attention^5–11^, and stimulus expectations^12,13^ all have been associated with changes in sensory representations. These changes may occur very early in the visual hierarchy^14^ and take the form of changes in sensitivity^10,11,15,16^, shifts in feature selectivity^1,4,17–21^, increases in baseline response^22–27^ useful for efficient selection^3,28^, and changes in the structure of stimulus-driven and noise correlations^2,29^.

However, flexible readout rather than change in sensory representation can be a behaviorally advantageous implementation of task demands. Although changing sensory representations can be beneficial, there can be associated behavioral costs to suppressing ignored features^30–32^ when these are actually relevant to behavior. In many dramatic demonstrations^33,34^, observers have been made blind to salient events when reporting about other aspects of a visual scene. This suggests a potential advantage to maintaining stable sensory representations and using flexible sensory readouts to enable adaptable behavior^35–37^.

To establish that a change in sensory representation between different task conditions is large enough to explain perceptual behavior, we can turn to linking models. Quantitative linking models^3,28,38–42^ connect measurements of cortical activity to behavior by modeling the presumed process by which sensory activity gives rise to perceptual behavior. Such linking models are explicit hypotheses and can be falsified if they are unable to quantitatively link change in sensory representations to behavior across different task conditions.

Here we use a linking model to study human reports of motion visibility and to understand whether sensory change or flexible readout implement this behavior. We first establish that observers can independently report about either the contrast (luminance difference between dark and bright dots) or motion coherence (percentage of dots moving in a coherent direction) of random dot patches while ignoring the other feature. We then extend a well-established linking model of human contrast perception^3,28,43–47^ to account for behavioral performance during these tasks. Because in individual cortical areas the response to motion visibility is mixed^48^, we allow the model to weight retinotopic areas according to their sensitivity to the two features. The critical step to understand behavioral flexibility was to measure blood oxygen-level-dependent (BOLD) signal while observers performed each discrimination task. If sensory representations changed enough, then a linking model with a fixed readout of sensory areas should be sufficient (i.e., that used the same weighting of cortical responses for both tasks). Implementing such a fixed-readout model shows that sensory changes alone are insufficient in magnitude to explain perception. Instead, in addition to the sensory change, a change in readout between different task conditions is necessary (i.e., a flexible readout). A benefit of flexible readouts is that sensory representations can retain information about the unattended feature. In line with this, we show that observers can re-map their reports unexpectedly.

## Results

### Perceptual sensitivity to motion visibility

**Fig. 1:**
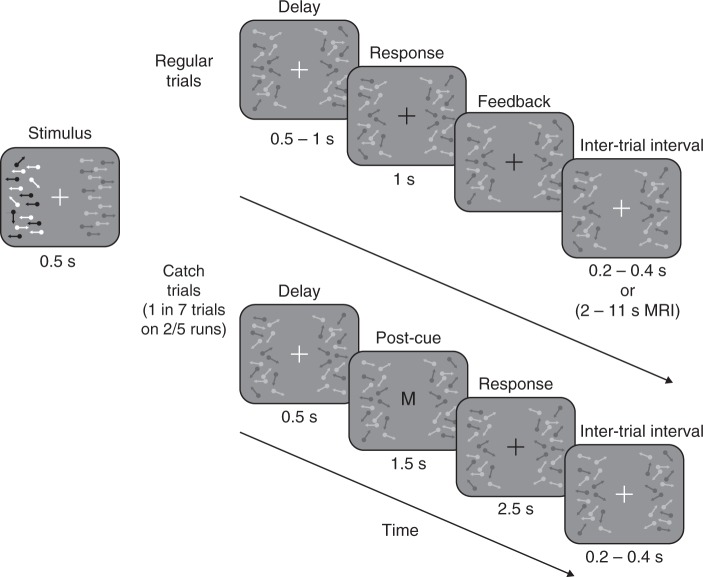
Behavioral task. Observers discriminated which of two random dot stimulus patches had higher contrast or coherence in different blocks of trials. Each block began with the word “contrast” or “motion” indicating that observers should report about contrast or coherence, respectively, and ignore the other feature. Between trials (Inter-trial interval) and during all but the Stimulus segment, the dot patches were presented at 25% contrast with incoherent motion. On each trial, both dot patches increased by independent base increments of contrast and coherence (+7.5, +15, +30, or +60% contrast; +15, +30, +45, or +60% coherence) for 0.5 s (Stimulus). In addition, for each feature one side was chosen independently to have an additional threshold-level increment, determined by a staircasing procedure. For regular trials, after a 0.5–1-s period (Delay), observers were asked to report which side contained the additional increment in contrast or coherence (Response) and were given feedback (Feedback). In a subset (Catch trials) of runs (2/5) on rare trials (1/7), the delay period was followed by a second cue (Post-cue), the letter “M” or “C”, indicating that the observers should prepare a response about the un-cued feature. Additional time was given to observers to make these decisions (post-cue period of 1.5 s, response window of 2.5 s) and observers did not receive feedback on catch trials

We characterized human perceptual sensitivity to the contrast and coherence of moving dots while observers had to report exclusively about one feature and ignore the other. We measured observers’ just-noticeable differences (JNDs) in image contrast or motion coherence between a pair of simultaneously presented random dot patches in a two-alternative forced choice task (Fig. 1). Each block of trials began with either the word “contrast”, indicating that observers should report which dot patch had higher contrast while ignoring differences in coherence, or “motion”, indicating the opposite. Each trial consisted of a 0.5-s base increment in the contrast and coherence of both dot patches (at all other times, the dot patches were kept visible at 25% contrast and 0% coherence). In addition to this base increment, a small additional increment near perceptual threshold was added to one side independently for each feature. Therefore, for every trial regardless of cueing condition there was a difference in both features between the two dot patches and each patch was equally likely to contain the additional increment. After stimulus presentation and a brief delay, observers reported which side had the higher magnitude of the cued feature and received feedback.

**Fig. 2:**
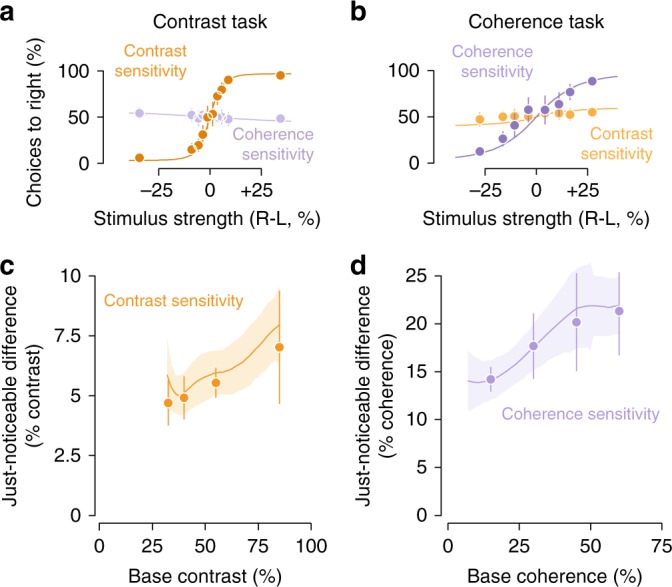
Perceptual sensitivity to contrast and motion coherence and fit of validation linking model. **a** Contrast task. The markers plot the average probability across observers and base stimulus strengths of indicating that the right dot patch had higher contrast or motion coherence while performing the contrast task, as a function of the difference in contrast (orange) or coherence (blue) between the two patches. Curves plot the predictions of the eight-area linking model using measurements made during passive viewing. These were fit to each individual observer’s behavioral data with a flexible readout, therefore fitting each task separately. **b** Coherence task, conventions same as **a**. **c** Markers plot the just-noticeable difference for contrast during that task estimated from a Weibull function fit for each base stimulus strength, averaged across observers. Curves indicate the average prediction of the eight-area linking model across observers. **d** Same as **c** for the coherence task. All markers indicate the mean and error bars the 95% confidence interval across observers. Curves indicate the mean model prediction across observers and shaded areas the 95% confidence intervals. Some error bars are hidden by the markers

Observers were able to report about each motion visibility feature independently. Collapsing across observers and base stimulus strengths, we found that observers were sensitive to the feature they were asked to report (dark orange, Fig. 2a, and dark purple, Fig. 2b) but insensitive to the features they were asked to ignore (light purple and light orange, Fig. 2a, b). The psychometric functions (circle markers in Fig. 2a) were well fit by cumulative normal distributions (not shown, average cross-validated $r_{{\mathrm{pseudo}}}^2$ = 87.7%). This suggests that observers’ decisions were consistent with a signal detection process in which two sensory representations were compared subject to Gaussian noise. Separating out sensitivity by base stimulus strength, we observed a proportional increase in JNDs (Fig. 2c, d) reminiscent of Weber's law. Weber’s law states that the slope of this relationship should be 1 on a log–log axis but we found slopes <1 for contrast, 0.44, 95% confidence interval (CI) [0.41 0.50], consistent with previous studies^3,49,50^, and 0.81, 95% CI [0.78 0.85] for coherence. Fitting a Weibull function on a subject-by-subject basis for base contrasts 32.5, 40, 55, and 85%, we found JNDs in contrast (Fig. 2c) to be 4.6%, 95% CI [3.8, 5.5], 4.8%, 95% CI [3.9, 5.8], 5.5%, 95% CI [4.9, 6.2], and 7.5%, 95% CI [5.3, 9.8], respectively. For base coherences 15, 30, 45, and 60%, we found JNDs in coherence (Fig. 2d) to be 14.2%, 95% CI [12.9, 15.5], 17.7%, 95% CI [14.3, 21.1], 20.2%, 95% CI [15.1, 25.3], and 21.3%, 95% CI [16.7, 25.9], respectively. Note that for contrast the base stimulus strengths are reported as the absolute value and not the relative increment from the 25% contrast and incoherent motion that was shown continuously throughout the experiment.

### Changes in cortical representation during task performance

**Fig. 3:**
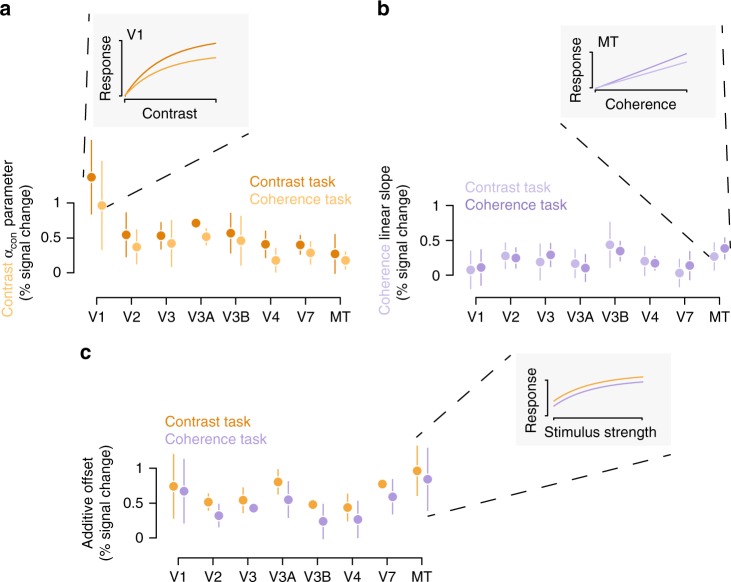
Cortical measurements during active viewing. Observers performed the behavioral task while hemodynamic responses in retinotopic visual cortex were measured. **a** The average across observers of the *α*_con_ parameter, a measure of contrast sensitivity, is shown for each task context (dark and light markers are the contrast and coherence task, respectively). Inset shows how the change in the parameter affects the change in the contrast response function for V1, ignoring any change in additive offset. **b** As in **a** for coherence sensitivity, as measured by the linear slope of the coherence response function (dark and light markers are coherence and contrast task, respectively) and inset shows the relationship for MT. **c** As in **a**, **b** for the *α*_task_ parameter, which absorbs additive offsets. Inset shows the additive offset shift for MT

We measured BOLD signal in retinotopically defined visual areas and found small changes in sensory responses when observers switched between reporting contrast and coherence (Fig. 3). Ten of the observers who performed the behavioral experiments repeated the task in the magnet. We used these measurements to examine how the contrast and coherence responses changed, either by multiplicative gain or additive offset, in each visual area (see “Methods”). For a majority of subjects, we found that when reporting about contrast, compared to reporting about coherence, the response to contrast in cortex showed a multiplicative gain (Fig. 3a). The average increase in *α*_con_ (Eq. 4) over areas and observers was 0.13% signal change/unit contrast, 95% CI [0.07, 0.19]. The direction of this effect was not always consistent. In V1 8/10 observers showed an increase; for V2 6/10; V3 7/10; V4 7/10; V3A 7/10; V3B 7/10; V7 5/10; MT 6/10. For the coherence response, we found no consistent change in the slope of the response function when reporting about coherence (Fig. 3b). The average over areas and observers was −0.02 % signal change/unit coherence, 95% CI [−0.08, 0.04]), though some individual areas like MT showed an increase. These changes were inconsistent across observers, in V1 6/10 observers showed an increase in the linear slope of the coherence response; V2 6/10; V3 6/10; V4 6/10; V3A 4/10; V3B 5/10; V7 6/10; MT 6/10. In some linking models, additive offsets have been shown to account for the perceptual benefits of selective attention^3,28^. We found that reporting about the stimuli, rather than passively viewing them, led to an additive offset in most visual areas (Fig. 3c). Average increase in *α*_task_ (Eq. 6) over areas and observers compared to passive viewing was 0.36% signal change, 95% CI [0.30, 0.44]. Additive offsets were slightly larger during the contrast task than the coherence task (Fig. 3c). Averaged over areas and observers this effect was a modest 0.07% signal change, 95% CI [0.01, 0.14]. In summary, we measured small changes in sensory response between task conditions and found that in some cortical areas contrast sensitivity increases when subjects perform the contrast task and coherence sensitivity increases when subjects perform the coherence task. While these changes are in the right direction to underlie task performance, a formal linking model is required to determine whether they are large enough to account for perceptual behavior.

### Linking model between cortical representation and perception

**Fig. 4:**
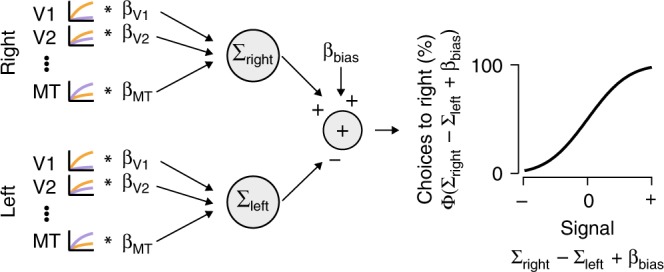
Readout linking model. The linking model simulates the cortical response evoked by each dot patch according to an existing framework^48^, which parameterized the contrast response function (orange curves) as a Naka–Rushton and the coherence response function (blue curves) as linear or a saturating exponential function. The model weights the cortical responses from each visual area (*β* values) evoked by the stimulus (Right or Left) according to the current task. The model then takes the difference between the signals evoked by each stimulus, plus a bias term (*β*_bias_) to account for any individual observer’s bias to choose one response over the other. To convert from this weighted signal to probability of choosing the patch on the right, the signal is passed through a cumulative normal distribution (curve on right). The linking model is analogous to probit regression with nonlinear input signals

We set out to build a linking model that could quantitatively predict behavioral performance from measurements of cortical sensory representation (Fig. 4). Once validated, such a model could then be used to assess whether the sensory changes we measured were large enough to explain behavioral performance in the task conditions. Linking models have been built for contrast discrimination tasks by assuming that higher contrast is detected by comparing the magnitude of cortical responses evoked by different stimuli, subject to some noise^3,28,43–46^. Behavioral sensitivity is determined by the ratio of response difference to the standard deviation of the noise, as in the classic signal detection measure *d’*. In our task, cortical responses are the result of stimuli that differ both in contrast and coherence. The linking model therefore needed to be able to differentiate which feature caused a difference in response. We reasoned that this could be accomplished by properly weighting visual areas according to their sensitivity to each stimulus feature. Our model took the form of a probit regression^51^ in which the difference in weighted response of visual areas to the two stimuli was computed and passed through the cumulative normal distribution to predict the probability of different choices (Fig. 4, see “Methods: Linking model” for full description).

Before evaluating such a model on the measurements of cortical activity during task performance (Fig. 3), we wanted to validate that such a linking model could in principle account for contrast and coherence discrimination. In previous work, we published measurements of contrast and coherence response in cortex while observers passively viewed the same random dot stimuli used here^48^. These measurements were used to quantify the shape of contrast and coherence responses across retinotopically defined visual areas using functional forms (Naka–Rushton for contrast and a saturating exponential form for coherence, see Eqs. 4 and 5). These passive-response data showed, for example, that V1–V4 are relatively more sensitive to changes in image contrast, whereas MT is more sensitive to changes in motion coherence. Using the functional forms measured during passive viewing, we simulated the trial-by-trial response of eight visual cortical areas, V1–V3, V4 (hV4), V3A, V3B, V7, and MT (hMT+), and modeled sensory readout on each trial as a task-dependent linear weighting of the population responses (Fig. 4). This resulted in a scalar response for the left and right stimulus patches (∑_right_, ∑_left_) on each trial. The observer’s decision about which side had the higher cued feature was modeled as a comparison between these two scalar responses (∑_right_–∑_left_) summed with a side bias (*β*_bias_). This scalar decision variable was subject to Gaussian noise as implied by the cumulative normal of the probit link function. We fit the parameters of the linking model using maximum likelihood estimation for each observer (8 cortical area weights × 2 task conditions + 1 bias parameter = 17 total parameters) using the average population response function parameters from Birman and Gardner^48^. For reference, these parameters describing sensitivity in different cortical areas are reported in “Methods”.

We found that the linking model based on the passive viewing BOLD data was a good fit for the behavioral measurements (curves, Fig. 2), capturing both the shape of the psychometric functions and the increase in JNDs with increasing base stimulus strength. To evaluate each model’s goodness-of-fit, we examined Tjur’s coefficient of determination (CD), a measure intended to be interpreted similarly to *r*^2^ for models of binary decisions^52^. To compare models, we computed cross-validated log-likelihood ratios (see “Methods: Model comparison”). We found across observers an average CD of 0.44, 95% CI [0.42, 0.45] reflecting that the model captured the sensitivity of human observers to differences in visibility across both task conditions (curves, Fig. 2a, b) as well as the reduced sensitivity at increasing base stimulus strength (curves, Fig. 2c, d). The fits shown are for the 8-area model, but we also tested a model with only the two areas with the highest contrast and coherence sensitivity, V1 and MT (2 cortical area weights × 2 task conditions + 1 bias parameter = 5 total parameters). We found a similarly good fit, $\log \left( {\frac{{{\cal{L}}_2}}{{{\cal{L}}_8}}} \right) = 7.38$, 95% CI [−3.09, 32.78]. The average CD of the 2-area model was also 0.44, 95% CI [0.43, 0.45].

**Fig. 5:**
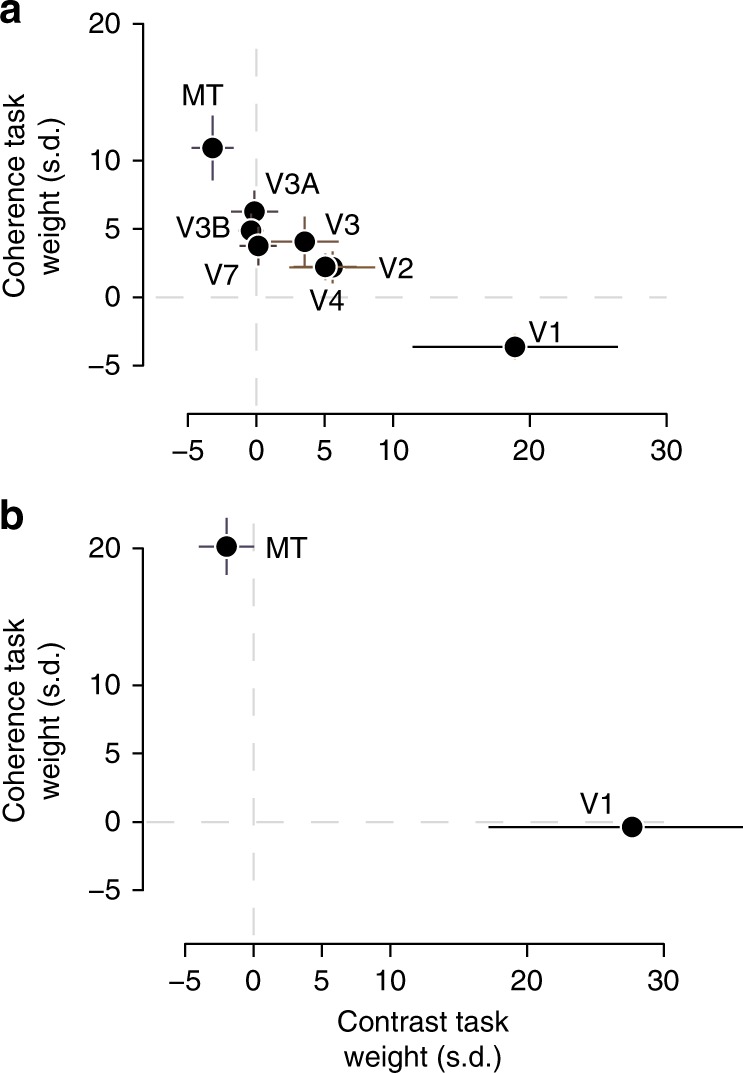
Cortical area weights. **a** The weights of the flexible-readout model fit to passive viewing data are shown for the contrast task (*x* axis) and coherence task (*y* axis) for the eight cortical areas we defined retinotopically: V1, V2, V3, V4 (hV4), V3A, V3B, V7, and MT (hMT+). **b** As in **a** but for the two-area model with only V1 and MT. All markers indicate the mean across observers and error bars the 95% confidence intervals

The linking model fit weights according to the relative sensitivity of each cortical area to contrast and coherence (Fig. 5). In the eight-area model, the contrast task weights (*x* axis, Fig. 5a) are proportional to how sensitive each area is to contrast relative to coherence: V1–V4 have positive weights, while only MT was given a negative weight. The negative weight on MT counteracts sensitivity to coherence in V1–V4 and ensures the linking model was insensitive to coherence when reading out contrast. The weights for the coherence task (*y* axis, Fig. 5a) behaved similarly, with MT getting the largest positive weight and V1 a slight negative one. A similar pattern was observed for a model with only areas V1 and MT (Fig. 5b) but with less negative weighting in the coherence readout.

**Fig. 6:**
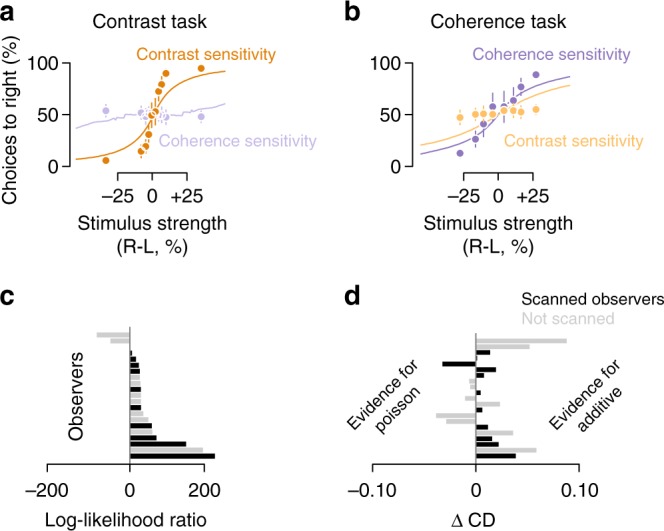
Poisson vs additive noise. **a**, **b** Same conventions as Fig. 2a, b, except curves indicate the prediction of a model incorporating Poisson-like noise in which variance scales equal to response strength. **c** The cross-validated likelihood ratio (difference in log-likelihood) between the additive and Poisson models is shown for each observer. **d** The change in Tjur’s CD comparing the additive models against the Poisson models, a measure analogous to *r*^2^. Observers are sorted as in **c**. In **c**, **d** evidence for the Poisson model is plotted toward the left and evidence for the additive model to the right

Using model comparison, we validated our linking model assumptions that sensory noise is additive and that observers had no dependency on choice history. Models based on single-unit variability often assume a Poisson-like noise^53^. We modeled an additive noise component because our model is based on population activity for which independent single-unit variability would be expected to average out. This choice of additive Gaussian noise was important. A model using Poisson noise, which increased with the average stimulus response magnitude, did not fit the data (Fig. 6). On average across observers, the additive model was a better fit compared to the Poisson model, $\log \left( {\frac{{{\cal{L}}_{{\mathrm{additive}}}}}{{{\cal{L}}_{{\mathrm{Poisson}}}}}} \right) = 43.58$, 95% CI [18.84, 77.93] (Fig. 6c) and improved CD by 0.01, 95% CI [0.00, 0.02] (Fig. 6d). A number of studies have found that observers performing psychophysical tasks are biased by previous choices even when those choices are uninformative for the current trial^54,55^. We tested for possible biases due to choice history (see “Methods”) but found that including these additional fit parameters caused the cross-validated log-likelihood to deteriorate, suggesting over-fitting, $\log \left( {\frac{{{\cal{L}}_{{\mathrm{original}}}}}{{{\cal{L}}_{{\mathrm{stay/switch}}}}}} \right) = 3.66$, 95% CI [0.31, 9.08]. Thus, model comparison was able to validate that choice history effects were negligible and that noise was best assumed to be additive rather than Poisson.

### Using the linking model to test fixed vs flexible readout

Having verified the linking model based on passive viewing data, we now asked whether the small changes in sensory representation which we measured during task performance could account for how perceptual sensitivity changed when observers switched task. If sensory changes were sufficiently large, then the readout could be fixed between task conditions. Such a fixed-readout model would only require a single set of cortical area weights with changes in perception accounted for only by changes in sensory responses.

**Fig. 7:**
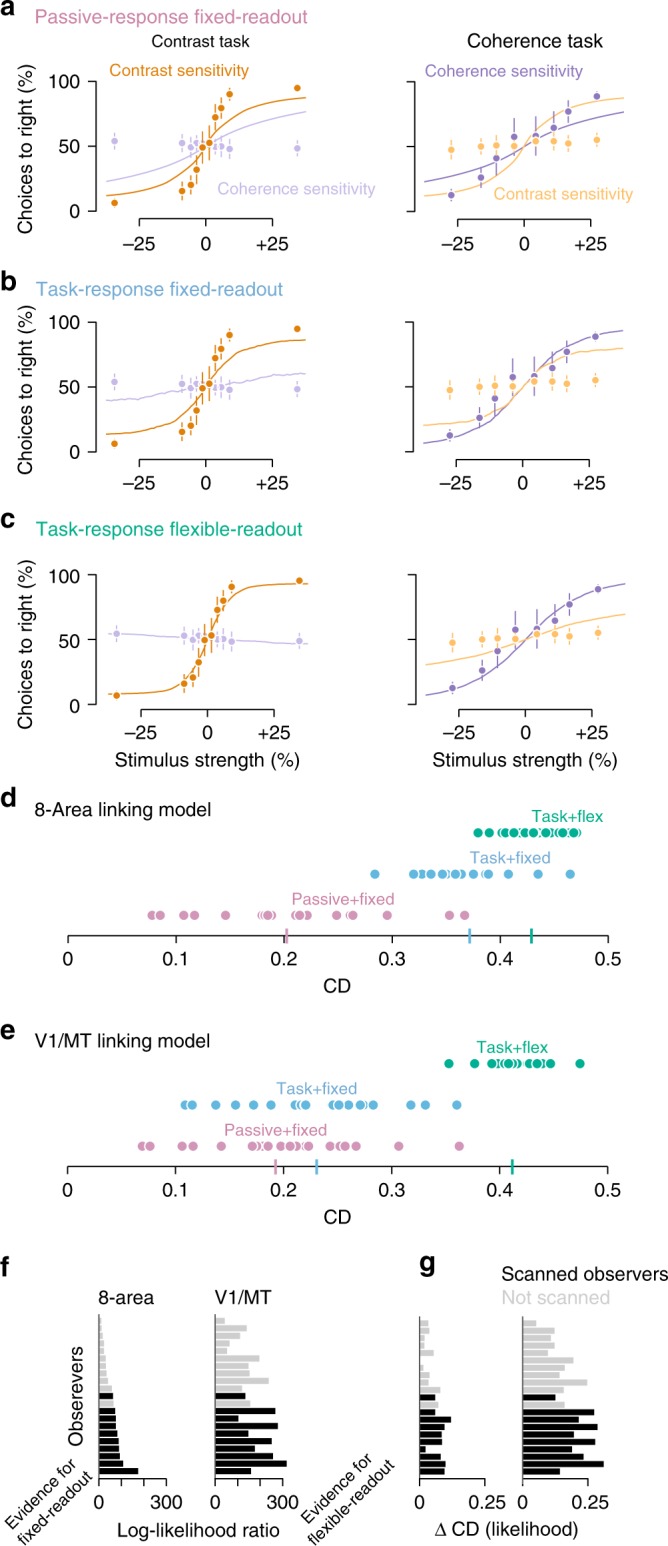
Comparing fixed- and flexible-readout linking models. **a**–**c** Same conventions as Fig. 2a, b, except curves plot: **a** the fit of the passive cortical response with a fixed readout, **b** the cortical response during task performance with a fixed readout, and **c** the cortical response during task performance with a flexible readout. The fixed-readout model forces any change in perceptual sensitivity to be the result of differences in sensory response between tasks by using only a single set of cortical readout weights (eight weights and one bias term). The flexible-readout model allows a different set of weights for each task condition (16 weights and 1 bias term). **d** Tjur’s CD for the models in **a**–**c**. Averages are shown as a bar on the axis. **e** Conventions as in **d** for the two-area models with only V1 and MT. **f** Model comparison of the cross-validated likelihood ratio (difference in log-likelihood) between the task-response fixed-readout and task-response flexible-readout models. Evidence for the fixed-readout model is plotted to the left and flexible-readout to the right (none of the fits are in favor of the fixed-readout model). **g** As in **f** for Tjur’s CD. Markers in **a**–**c** indicate the average across observers. Markers in **d** and **e** indicate individual observers. Error bars are the 95% confidence intervals. Some error bars are hidden by the markers

As a baseline for comparison, we first fit the fixed-readout model on the sensory responses measured during passive viewing. By definition, there are no sensory changes between task conditions so this passive-response fixed-readout model can only produce behavior that is intermediate between the two tasks. That is, it is sensitive to both contrast and coherence (Fig. 7a, orange/yellow contrast curves and blue/purple coherence curves are not flat) and cannot switch sensitivity between the two tasks (Fig. 7a, curves for left and right panels are identical). The CD and likelihood of the passive-response fixed-readout model provide a lower bound on the possible explainable variance (Fig. 7d, e).

Fitting the fixed-readout model to sensory responses measured during task performance showed that, while changes in sensory response could account for a substantial amount of the behavioral performance, the changes were insufficiently large to fully explain task performance. This task-response fixed-readout model achieved a better fit of the behavioral data than the passive-response fixed-readout model (Fig. 7d, e, compare magenta and blue points) thus quantifying how much the sensory changes reported above can account for behavioral performance. Indeed, the task-response fixed-readout model was better able to capture differences in behavior between the contrast and coherence task (Fig. 7b, compare curves for left and right columns). However, the linking model failed to completely capture the ability of subjects to change their perceptual sensitivity to contrast and coherence between the two tasks. In the contrast task, the contrast sensitivity curve (orange, Fig. 7b) does not match the sensitivity of the observers and the model predicted a weak bias for coherence (light purple, left panel of Fig. 7b) that the subjects did not show. In the coherence task, the coherence performance was reasonably well matched (purple curve, right panel), but the model predicted strong bias from contrast (orange curve).

Rather than rely only on changes in sensory representation between tasks, a linking model that could read out responses from visual areas differently between tasks was better able to fit the behavioral performance. We tested this task-response flexible-readout model by allowing the weights for different visual areas to change between tasks while still using the sensory responses measured during task performance. This model provided reasonable fits to the behavioral data (Fig. 7c), capturing the performance during the contrast task (left column), although it did predict slightly more bias to contrast during the coherence task than the observers displayed (orange curve, right panel). Because the fixed-readout and flexible-readout models had different numbers of parameters (fixed readout = 9 parameters, flexible readout = 17), it was critical to evaluate the models with a cross-validated metric. We found that for the task-response measurements the flexible-readout model was a far better fit than the fixed-readout model (Fig. 7f, g), $\log \left( {\frac{{{\cal{L}}_{{\mathrm{Flexible}}}}}{{{\cal{L}}_{{\mathrm{Fixed}}}}}} \right) = 60.16$, 95% CI [44.90, 77.75], difference in CD, 0.06, 95% CI [0.04, 0.07]. Note that observers who we measured physiology for (black bars, Fig. 7f, g) show a larger improvement in model fit compared to the other observers, which we attribute to an effect of increased training (see Supplementary Note 1).

As additive offsets have been used with a fixed readout to explain differences in behavioral performance with spatial attention^3,28^, we also tested an efficient selection model that weights responses according to their magnitude. We found that this model could not explain the behavioral performance (Supplementary Note 2).

### Behavioral evidence for a flexible readout

One advantage to keeping sensory representations relatively stable is that observers can maintain information about unattended features. To measure whether observers could recall unattended information, we included “catch” trials in the behavioral task. In catch trials, observers were shown a post-cue after stimulus presentation, which indicated that they should report about the un-cued feature (bottom time line, Fig. 1). Observers made these reports despite the stimulus having already been presented and despite having already had 0.5 s to prepare their response for the main task. We were able to ensure observers did not split their attention by keeping the main task at perceptual threshold, making catch trials rare, and not providing feedback (Supplementary Note 3).

**Fig. 8:**
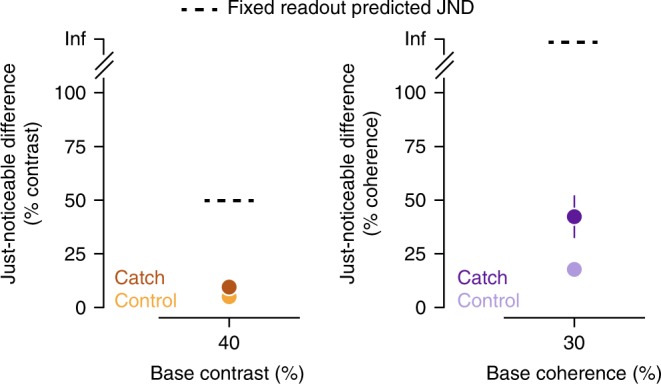
Perceptual sensitivity on catch trials. Just-noticeable differences (JNDs) for contrast (left) and coherence (right) are shown for the regular (control, light colors) and catch (dark colors) trials during runs that included catch trials. Dashed lines indicate the predicted JNDs for catch trials for the fixed readout model. Markers indicate the average across observers and error bars the 95% confidence intervals, some error bars are hidden by the markers

During catch trials, we found that observers were less sensitive to the un-cued motion visibility features compared to when they were cued, but nevertheless they maintained significant information about the unattended features. Observers’ JNDs were larger on the catch trials both for the contrast task (average Δ JND = +5.30% contrast, 95% CI [+3.83, +7.22]) and coherence task (Δ JND = +45.84%, 95% CI [+26.17, +98.23]) (Fig. 8). These averages (and subsequent analysis) exclude 4/21 and 1/21 observers for the coherence and contrast tasks, respectively, because they could not perform the task and their JNDs were not measurable (i.e., their JND was more than what could be displayed on the screen).

If observers had a fixed readout that could not switch to the ignored feature during catch trials, then they would be forced to use the wrong readout and performance would be extremely poor. We found that this fixed-readout model predicted much higher JNDs than measured and therefore could not account for catch trial behavior. That is, we used the task-response flexible-readout model to compute the expected JND on catch trials assuming that observers were unable to switch the readout to the post-cued feature. For example, for the contrast task in which the catch trials required making a coherence judgment, we used the cortical readout weights for the contrast task (from Eq. 7: *β*_V1,_ *β*_V2_,…) and vice versa. This model underestimated human performance on catch trials (dashed lines, Fig. 8). On contrast catch trials (i.e., post-cued trials when observers reported about contrast, during a run where the main task was coherence), the model predicted JND of 56.9%, 95% CI [33.8, 80.1], but the average observer had a JND of only 10.1% contrast, 95% CI [7.5, 12.7]. On coherence catch trials, the model predicted that observers would be incapable of performing the task, but the average observer JND was 40.8% coherence, 95% CI [32.4, 49.2].

Instead, we found that a better explanation for catch trial behavior came from a readout that could dynamically change within trials but incurred an additional cost for maintaining sensory information in working memory. This cost could be due to a drop in the signal-to-noise of the sensory representation, perhaps due to responses degrading over time. We estimated the cost by dividing the thresholds measured during catch trials by the thresholds measured during regular trials. This approach suggests that on average *σ* increased (or responses degraded) for coherence by a factor of 3.44, 95% CI [2.46, 5.90] and for contrast by 2.21, 95% CI [1.87, 2.66]. The overlap in estimates suggests a single cost, i.e., the change from a discrimination task to a working memory task, might govern the change in performance for both tasks; averaging the increase in noise gives an estimated reduction in sensitivity of 2.83, 95% CI [2.31, 4.17]. Thus a model that allows rapid re-weighting, combined with a fixed cost for using working memory, can explain behavioral performance for both contrast and coherence catch trials.

## Discussion

We found that observers were able to independently judge the visibility of patches of moving dots based on either their contrast or coherence. Concurrent measurements of BOLD activity showed that there were small changes in sensory representations during task performance. Cortical responses were somewhat more sensitive to contrast during contrast discrimination and, in some areas like MT, more sensitive to coherence during the coherence task. Our analysis with a fixed-readout linking model showed that these changes could account for some but not all of the behavioral performance. A flexible readout of sensory representations was necessary to fully capture the behavior. Keeping representations relatively stable should allow observers to retain information about unattended features, and we found that during catch trials this was the case. Our results highlight the importance of using models that quantify the link between cortical representation and perception.

We manipulated the contrast and coherence of random dot motion stimuli because of the extensive existing knowledge of how neural representations of these features are related to visual perception^41,43,44,46^ and because their similar representation in cortex^48^ suggests that changing the representation of one will necessarily affect the other. Contrast, the average difference between bright and dark, and coherence, the percentage of dots moving in the same direction, both control the visibility of motion. Human cortical visual areas are known to be sensitive to these properties such that an increase in visibility results in monotonically increasing responses throughout the visual cortex^44,48^. For observers to judge these two features independently, their sensory representations need to be separated according to context, a step that existing linking models built for single features have not had to contend with.

The computational steps from sensory representation to perception have been well characterized for contrast discrimination. In these linking models, an observer’s choice is computed by comparing the evoked neuronal responses to different stimuli. Individual neurons exhibit monotonically increasing responses to contrast^56^, with different parameterizations that can be pooled into a population response^57^. Such population responses to contrast are well indexed by BOLD signal in human visual cortex^44,58^. Linking models have been shown to account for BOLD signal measurements and perceptual responses during contrast discrimination tasks^43^, predict changes in these measures during surround masking^47^ and detection^27^, and have been used to describe the selection of signals from attended locations^3,28^.

Our model extends a linking model of contrast discrimination^3,28,43–47^ to simultaneous judgments of contrast and coherence. To separate the intertwined sensory representations of these features, we allowed a linear weighting of cortical areas. The weights fit by the model confirmed that the bulk of information for these simple perceptual decisions was available in V1 for contrast perception and MT for coherence. This matches with previous results implicating monkey MT in judgments about motion^41^. But the weights also revealed that other areas could play an important role in perception by suppressing correlated signals about un-cued features in the readout. Our linking model is also specific to the random dot stimulus we chose. Changing the dot density^59^ or aperture size^60–62^ can result in decrements or zero response to increasing coherence, which would necessitate a linking model specific to those stimulus properties. We chose our stimulus size, dot density, and dot speed with these concerns in mind (for additional discussion of how stimulus properties affect the coherence response, see Birman and Gardner^48^).

The linking model we developed held only if sensory noise was modeled as additive but not if variability increased in proportion with sensory response^53^. Additive noise appears repeatedly in the literature using linking models^3,28,43,63^, in purely psychophysical approaches^49,64^, and in measurements of population activity from voltage-sensitive dyes^65^. In our results, the Poisson noise model failed because it combined increasing noise with response functions that saturate^48^; either of which alone predicts the cumulative normal form of the psychometric functions and a Weber law-like effect at increasing base stimulus strengths. This result suggests that the noise that limits perceptual behavior is not the individual variability in firing rate of single neurons, which presumably is averaged out across a population, but a correlated source of variability, which is not dependent on response amplitude.

Our results demonstrate that sensory change due to attention does not transform the sensory representation directly into a form that can be used to drive motor responses. Instead, switching from reporting one stimulus property to another must change the readout (i.e., weighting of connections), which may begin to occur in sensory cortices^66^ but must also extend beyond them. One possible role for the response gain is that it works together with changes in readout, acting, as we calculated, as a weak form of sensory enhancement. Recent theoretical and experimental results suggest that such changes might improve the ability of a linear readout to differentiate between stimulus-driven and internal signals^16,67,68^. These changes match with our finding that noise limiting perceptual sensitivity is due to correlated internal variability. Sensory changes might also drive responses to be more aligned with the readout dimension^69^, effectively working together.

Although for our task the scale of sensory changes provided only a partial explanation for context-dependent behavioral reports, this need not always be the case. In the literature on visual attention, there are many examples of changes in sensory representation as a result of task demands^1–11^. We interpret these results and our own as falling within a continuum where task demands are implemented by complementary changes in sensory representation and sensory readout. Sensory effects that can alone account for behavioral changes would be at one end of this continuum. For example, measurements of changes in spatial tuning^1,70,71^ may underlie bottom–up biases in spatial perception^72^, additive shifts in response^22,25,26^ can be used by efficient selection mechanisms to account for perceptual threshold enhancement^3,23,28^, and changes in correlation structure during focal spatial attention^2^ can be sufficient to explain changes in perceptual sensitivity^29,39,69^. These spatial attentional effects may reflect the combination of a fixed sensory readout combined with changes in representation, which select^3,28^ and align^69^ relevant signals while suppressing others. Similar mechanisms may also play a role in some kinds of feature-based attention, especially search^73^.

Our results suggest that judgments of motion visibility rely on both a context-dependent readout and changes in sensory representation, putting our task in a different part of the continuum described above. Relying on flexible readout could help maintain adaptability in the face of uncertain task demands. It is possible that given enough time and task consistency observers could have shifted their cortical implementation to solve our task using a fixed readout. This could be done by learning to pre-select relevant sensory representations, saving computational cost and speeding decision making. Similarly, sensory representations may be kept stable for visual features that are relevant for a variety of behaviors. For example, scene gist is known to survive inattention, both perceptually^74^ and as decodable information from BOLD signal measurements of visual cortex^37^.

How the human brain implements task demands may depend not only on the form of sensory representation, the precise task demands, and the extent of learning but also on the associated computational costs^75^. Flexible readout might be implemented by parts of prefrontal cortex which re-represent visual information in a context-dependent manner^35^ using dynamical properties that can selectively integrate different features of sensory stimuli^36^. Engaging these mechanisms requires resources to represent and process aspects of sensory stimuli that may not be behaviorally relevant. Changing sensory representations and using a fixed readout may instead reflect a computationally efficient solution where the visual system no longer has to contend with representing irrelevant stimulus information. In general, the complimentary mechanics of sensory change and change in readout are both essential tools for the human brain, allowing us to meet the demands imposed by daily life where constant shifts in attention are necessary to achieve our goals.

## Methods

### Observers

In total, 29 observers were subjects for the experiments. All observers except one (who was an author) were naive to the intent of the experiments. Eight observers were excluded during initial training sessions due to inability to maintain appropriate fixation (see “Eye tracking” below). All of the remaining 21 observers (13 female, 8 male; mean age 28 years; age range 18–55 years) performed the motion visibility behavioral experiment outside of the scanner. Observers performed up to 6 1-h sessions on separate days for an average of 2467 trials each (range 1167–3652, standard deviation 497). Ten of the observers (7 female, 3 male; mean age 26 years; age range 19–36 years) repeated the motion visibility experiment inside the scanner. Observers were scanned in 2 90-min sessions, each consisting of 8 7-min runs, and a third 1-h scan, which included retinotopy and anatomical images. Procedures were approved in advance by the Stanford Institutional Review Board on human participants’ research, and all observers gave prior written informed consent. Observers wore corrective lenses to correct their vision to normal when necessary.

### Hardware set-up for stimulus and task control

Visual stimuli were generated using MATLAB (The Mathworks, Inc.) and MGL^76^. During scanning, stimuli were displayed via an Eiki LC-WUL100L projector (resolution of 1920 × 1080, refresh-rate of 100 Hz) on an acrylic sheet mounted inside the scanner bore near the head coil. Visual stimuli were viewed through a mirror mounted on the head coil and responses were collected via a magnetic resonance imaging-compatible button box. Outside the scanner, stimuli were displayed on a 22.5 inch VIEWPixx LCD display (resolution of 1900 × 1200, refresh rate of 120 Hz) and responses collected via keyboard. Output luminance was measured for both the projector and the LCD display with a PR650 spectrometer (Photo Research, Inc.). The gamma table for each display was dynamically adjusted at the beginning of each trial to linearize the display luminance such that the full resolution of the 8-bit table could be used to display the maximum contrast needed. Other sources of light were minimized during behavior and scanning.

### Eye tracking

Eye tracking was performed using an infrared video-based eye-tracker at 500 Hz (Eyelink 1000; SR Research). Calibration was performed throughout each session to maintain a validation accuracy of <1 deg average offset from expected using either a 10-point or 13-point calibration procedure. Trials were canceled on-line when an observer’s eye position moved >1.5 deg away from the center of the fixation cross for >300 ms. During training and before data collection, observers were excluded from further participation if we were unable to calibrate the eye tracker to an error of <1 deg of visual angle or if their canceled trial rate did not drop to near zero. After training, canceled trials consisted of <0.1% of all trials. Owing to technical limitations, eye tracking was not performed inside the scanner.

### Stimulus

Motion stimuli consisted of two patches of random dot stimuli flanking a central fixation cross (1 × 1 deg). The random dot stimulus patches were rectangular regions extending from 3.5 to 12 deg horizontal and from −7 to 7 deg vertical on either side of the fixation cross. Each patch was filled with 21 dots deg^−2^, 50% brighter, and 50% darker than the gray background (300 cd m^−2^ in the scanner and 46 cd m^−2^ during behavior). All dots moved at 6 deg s^−1^ updated on each video frame. Motion strength was adjusted by changing motion coherence: the percentage of dots that moved in a common direction with all other dots moving in random directions. Dots were randomly reassigned on each video frame to be moving in the coherent or random directions. Both patches maintained a constant baseline in between trials of 25% contrast and incoherent motion. To minimize involuntary eye movements, the coherent dot motion direction was randomized to be horizontally inward or outward from fixation on each trial, such that the two patches moved in opposite directions.

### Contrast and coherence tasks

Observers performed a two-alternative forced choice judgment about the visibility of the two dot patches (Fig. 1). At the start of each run, observers were shown the word “contrast” or “motion” cueing them to report which side had the higher contrast or motion coherence, respectively. Each run began with a 5-s baseline period during behavioral measurements or 30 s during scanning (25% contrast, 0% coherence) to allow time for adaptation to occur. Trials consisted of a 0.5-s increment in either or both the contrast and motion coherence of the dot patches, a variable delay of 0.5–1 s, and a response period of 1 s. The dot patches then returned to baseline for an inter-trial interval of 0.2–0.4 s randomly sampled from a uniform distribution (2–11 s, sampled from an exponential distribution during scanning). The base stimulus strength increments were chosen to be +7.5, +15, +30, and +60% contrast above the baseline 25% contrast and +15, +30, +45, and +60% coherence above the baseline 0% coherence. On every trial, one dot patch was chosen as the target for contrast and incremented by an additional small delta, and the same was done independently for coherence. The target increment for the uncued feature was randomly chosen from [0.0, 1.8, 2.5, 3.5, 4.9, 6.9, 9.5, 13.3, 18.5%] for contrast and from [0.0, 5.0, 6.9, 9.6, 13.4, 18.6, 25.9, 36.1, 50.2%] for coherence. The relevant target increment was chosen by a PEST staircase^77^ to maintain ~82% correct on the cued task for each base strength (4 base strengths × 2 task conditions = 8 total staircases). Observers indicated with a button press which side contained the delta increment of the cued feature. An observer would be at chance performance if they reported on the wrong feature. Staircases were initialized on the first run (after training) at 25% and 85% for contrast and coherence, respectively. The staircases were maintained across sessions, but the step size was reset to one third the threshold every third run to allow for long-term fluctuation. Before data collection, observers trained on the task until their performance at all base stimulus strengths was measurable (i.e., their threshold converged to <1 minus the base strength), on average 1 h of training. Behavioral runs lasted 4 min and observers took breaks as needed. Observers performed up to 6 1-h sessions of behavioral runs spanning multiple days.

On a subset of the motion visibility experiment runs (two of every five runs), observers were occasionally asked to report about the non-cued feature (trial probability 1/7, randomized). We refer to these as catch trials. Stimulus presentation occurred as normal on catch trials but after stimulus presentation and a fixed 0.5 s delay, a letter replaced the fixation cross to indicate that the observer needed to recall and respond about the un-cued feature. The length of the delay periods in both catch and regular trials (0.5 s and 0.5–1 s, respectively) were chosen to ensure observers could not rely on iconic memory to complete the task^78^ and to avoid observers getting into a rhythm and responding before the post-cue could appear. On contrast runs, the post-cue letter was an “M” indicating that observers should recall about motion coherence and on coherence runs a “C” to indicate contrast. To improve our statistical power in estimating perceptual sensitivity during catch runs, we used a single base stimulus increment: +30% contrast and +40% coherence. These base increments were used both for catch and regular trials on these runs.

### Behavioral data analysis

To assess whether the perceptual data could be well characterized by a signal detection framework we tested the fit of cumulative normal distributions to the measured psychometric functions. We collapsed data from all observers across the four base stimulus strengths and separated trials in which observers discriminated contrast or coherence. We binned data according to the difference in stimulus strength for each task and computed the probability of making a rightward choice in each bin (filled circles, Fig. 2a, b). We fit the binned data with a cumulative normal distribution (three parameters: the mean, *μ*, standard deviation, *σ*, and a lapse rate, *λ*, which scaled the function so that it spanned the range $\frac{\lambda }{2}$ to $1 - \frac{\lambda }{2}$) and evaluated the cross-validated fit on a held-out observer using the pseudo *r*^2^:

1 $$r_{{\mathrm{pseudo}}}^2 = 1 - \frac{{{\mathrm{log}}(L_{{\mathrm{model}}})}}{{{\mathrm{log}}(L_{{\mathrm{null}}})}}$$

where *L*_model_ is the likelihood of the model given the data and *L*_null_ is the likelihood of an intercept-only model.

### JND (threshold) estimation

To assess perceptual sensitivity, we obtained JNDs (or thresholds) by fitting a Weibull function to each observer’s data using maximum likelihood estimation:

2 $$P_{{\mathrm{correct}}}(x) = \gamma + (1 - \gamma - \lambda )(1 - e^{ - (\frac{x}{\tau })^\beta })$$

where *x* is the difference in signal (either contrast or coherence) between dot patches, *γ* is the guess rate, *λ* is the lapse rate, *β* controls the slope of the function, and *τ* the value of *x* at which the function reaches 63% of its maximum. For this two-alternative forced choice task, the guess rate was 50% while threshold (when *d’* = 1) corresponds to ~76% correct. In total, we fit 12 Weibull functions for each observer: 8 for the contrast and coherence task (4 base strengths × 2 task conditions), 2 for the cued tasks in catch runs (1 base strength × 2 tasks), and 2 for the catch trials (1 base strength × 2 tasks).

### Cortical measurement during task performance

We measured how contrast and coherence response functions changed in gain or offset compared to passive fixation in different retinotopically defined cortical visual areas as ten observers performed the contrast or the coherence discrimination task. Our general strategy was based on previous work^48^ in which we have shown that the relationship between contrast or coherence and BOLD response can be independently parameterized with functional forms, as described below. The analysis proceeded in the following steps. We first used population-receptive field measurements^79^ to determine the location of cortical visual areas in each individual subject. We then took the timeseries of data averaged across each visual area (for each hemisphere and subject) and performed an event-related analysis to compute the average response to the stimulus presented in the contralateral visual field for each of the 16 combinations of base contrast and coherence and 2 task conditions. We computed the amplitude of response by fitting these event-related responses to a canonical hemodynamic response measured during passive viewing. We had at least 42 measurements (21 repeats in 2 hemispheres) of each base stimulus combination for each task condition in each subject. Consistent with our overall conclusion of flexible readout, comparing these response magnitudes directly between conditions showed weak if any change between conditions. The 95% CI of the differences between tasks included zero for almost all conditions (amplitudes were higher during the contrast task compared the coherence task for 4/16 conditions, averaging over observers). This analysis does not separate out the independent effects of contrast and coherence across task conditions. So, to gain statistical power and to establish how these BOLD responses reflect difference in contrast and coherence response between task conditions, we used the response magnitudes to scale and shift the contrast and coherence response functions, originally based on data from passive viewing. These 6 parameter fits (2 gain parameters and 1 offset parameter for each of the 2 task conditions) were based on ~672 (16 base contrast and coherence conditions × 42 repeats) trial measurements, which provided sufficient statistical power and are reported in the main results. Note, for one subject the contrast and coherence values in the conditions differed: only 12 out of 16 conditions were run, and with slightly different contrast and coherence values, we were still able to fit the population response function models to this smaller dataset.

All BOLD imaging and data analysis procedures including imaging protocol, preprocessing, data registration across sessions, retinotopic definition of visual areas using population-receptive field measurements, and extraction of mean timeseries from each visual area followed procedures described in detail in Birman and Gardner^48^. Briefly, visual area mapping and cortical measurements were obtained using a multiplexed sequence on a 3-Tesla GE Discovery MR750 (GE Medical Systems) with a Nova Medical 32-channel head coil. Functional images were obtained using a whole-brain T2*-weighted two-dimensional gradient-echo acquisition (field of view (FOV) = 220 mm, repetition time = 500 ms, echo time = 30 ms, flip angle = 46 deg, 7 slices at multiplex 8 = 56 total slices, 2.5 mm isotropic). In addition, two whole-brain high-resolution T1-weighted 3D BRAVO sequences were acquired (FOV = 240 mm, flip angle = 12 deg, 0.9 mm isotropic) and averaged to form a canonical anatomical image, which was used for segmentation, surface reconstruction, session-to-session alignment, and projection of data onto a flattened cortical surface. Preprocessing was performed using mrTools^80^ and included linear trend removal, high pass filtering (cutoff of 0.01 Hz), and motion correction with a rigid body alignment using standard procedures^81^. Visual cortical areas V1–V4, V3A/B, V7 (IPS0), and MT (hMT+) were identified using the population-receptive field method^79^ and standard criteria^82^. Average time courses were obtained for each cortical visual area by averaging the top 25 task-responsive voxels per area. As documented in Birman and Gardner^48^, repeating the analysis using all voxels, the top two voxels, or all voxels weighted by their population-receptive field overlap with the stimulus results in a change in the signal-to-noise in the data but did not change the relative sensitivities across cortical areas.

To compute event-related responses, we assumed that overlapping hemodynamic events sum linearly, an assumption that has been validated explicitly for visual responses^83^. We used a randomized inter-trial interval to avoid cognitive^84^ and hemodynamic^85^ anticipatory effects and to increase the efficiency of our design^86,87^. As violations of linearity have been noted with shorter inter-trial intervals, we chose a mean inter-trial interval of 6 s, sampled from an exponential with a range of 2–11 s, intended to minimize the overlap in the main positive lobe of the hemodynamic response between different events. Moreover, we used a balanced design in which each trial was equally likely to be followed by a trial with any of the other base stimulus strengths to minimize any systematic misestimation. We confirmed that the probability of each condition being followed by any other was roughly equal, i.e. *χ*^2^(*r*, 15) > 0.05, where *r* was the test statistic computed by comparing the distribution of trial types following each individual trial type against a uniform distribution. No catch trials were run during scanning.

We computed event-related responses for each trial type using a finite-impulse response model^84^. We assumed each combination of different base strengths for contrast and coherence evoked a different hemodynamic response and responses that overlapped in time summed linearly. Because each visual stimulus was lateralized in one half of the visual field, we assumed that they evoked a response only in contralateral retinotopic areas. There were four base increments for contrast (+7.5, +15, +30, and +60%) and four base increments for coherence (+15, +30, +45, and +60%), which were independently manipulated, resulting in 32 total conditions (4 contrast × 4 coherences × 2 task conditions). To model these data, we used the following equation:

3 $$y = X\beta + {\it{\epsilon }}$$

where *y* was an *n* × 1 column vector (*n* = number of volumes) containing the measured hemodynamic response for one hemifield of one visual area in a single observer. *X* was an *n* × (*k* × *c*) stimulus convolution matrix (*c* = number of conditions, *k* = length in volumes of hemodynamic response to calculate), *β* was a (*k* × *c*) × 1 column vector to be estimated, and *ϵ* the residual variance (assumed to be 0 mean Gaussian). Each block of *k* columns in *X* corresponded to one of the *c* conditions. These blocks contained a one in the first column at the starting volume of each occurrence of a trial of that condition and zeroes elsewhere. Each of the subsequent *k* columns was then shifted downwards by one to form a Toeplitz matrix for that condition. In total, *X* had *n* rows, equal to the length of the BOLD timeseries (for most observers, *n* was 13,184), and 2592 columns (*k* = 81 × *c* = 32, where *k* was chosen to compute 40.5 s of response and the *c* conditions were the 4 contrast base strengths × 4 coherence base strengths × 2 tasks). By computing the least-squares estimate of the column vector *β*, we obtained the estimated event-related response to each condition accounting linearly for overlap in time. On every trial, one dot patch was at a base strength and one had an additional increment. To equate difficulty throughout the task, we allowed the additional increments to vary continuously via staircasing. To simplify the estimation problem and to improve statistical power, we rounded the base + increment values to the nearest base strength. The choice of number of volumes of response *k* to compute did not change the result as long as it was sufficiently large to capture the full hemodynamic response. The Pearson’s correlation of the first 41 volumes between an analysis with *k* = 41 (20.5 s of response) and *k* = 81 (40.5 s of response) was *r* = 0.97. Because we randomized trial presentation, we assessed multicollinearity by checking that the stimulus convolution matrices (see below) were full rank and that the off-diagonal elements of the covariance matrix were small (<0.1% of off-diagonal elements were >10% of the on-diagonal elements).

To obtain a response magnitude, we fit a scaled canonical hemodynamic response function to the event-related responses. We used a canonical hemodynamic response function that was measured in previous work when observers passively viewed the same stimulus^48^. This function took the form of a difference-of-gamma function whose maximum amplitude was set to one. We fit a single magnitude per condition, which scaled this canonical function to minimize the sum of squared error between the event-related response and the scaled canonical function. For each condition (4 contrast base strengths × 4 coherence base strengths × 2 tasks), this gave us a scalar response amplitude for the evoked activity in each cortical area.

The response magnitudes for each contrast, coherence, and task condition were next used to estimate how population response functions for contrast and coherence in different visual areas changed in gain and offset during task performance. In our previous work, we parameterized the population response to contrast as a sigmoid function:^56^

4 $$R_{{\mathrm{con}}}(s_{{\mathrm{con}}}) = \alpha _{{\mathrm{con}}}\left(\frac{{s_{{\mathrm{con}}}^{1.9}}}{{s_{{\mathrm{con}}}^{1.6} + \sigma ^{1.6}}}\right)$$

where *α* was the maximum amplitude and *σ* controlled the shape of the function. The exponents in the function were chosen according to previous work^43^. The population response function to coherence was parameterized to be a saturating nonlinear function:

5 $$R_{{\mathrm{coh}}}(s_{{\mathrm{coh}}}) = \alpha _{{\mathrm{coh}}}\left(1 - e^{\frac{{s_{{\mathrm{coh}}}}}{\kappa }}\right)$$

where the parameter *κ* controls the shape of the function by setting the point at which the exponential function reaches 63% of its maximum and *α*_coh_ controls the amplitude. Large values of *α*_coh_ combined with large values of *κ* make this function approach linear in the range [0 1] in which the stimulus strength *s*_coh_ is bounded. Because *α*_coh_ and *κ* are not interpretable on their own, we instead report the linear slope of the coherence response functions as a measure of sensitivity (see Birman and Gardner^48^, for rationale).

We fit the population response functions for each cortical area to the 32 measurements of response magnitude (4 base contrasts × 4 base coherences × 2 task conditions) during task performance:

6 $$R_{{\mathrm{area}}}(s_{{\mathrm{con}}},s_{{\mathrm{coh}}}) = R_{{\mathrm{area,con}}}(s_{{\mathrm{con}}}) + R_{{\mathrm{area,coh}}}(s_{{\mathrm{coh}}}) + \alpha _{{\mathrm{task}}}$$

We added the *α*_task_ parameter to fit additive offset while allowing the *α*_*con*_ (Eq. 4) and *α*_coh_ (Eq. 5) parameters to change to fit multiplicative gain. The parameters for the response functions were initialized according to the passive viewing data in Birman and Gardner^48^ with the *σ* and *κ* parameters held constant such that response functions maintained their shape. For reference, the initial *α*_con_ parameter in V1 was 1.68, V2: 0.69, V3: 0.63, V4: 0.61, V3A: 0.35, V3B: 0.24, V7: 0.32, and MT: 0.22. The initial slope of the coherence response function in V1 was 0.07% signal change/unit coherence, V2: 0.16, V3: 0.18, V4: 0.11, V3A: 0.25, V3B: 0.14, V7: 0.20, MT: 0.34. For each cortical area, there were six free parameters (3 parameters × 2 task conditions) fit by minimizing the sum of squared error using the MATLAB function *lsqnonlin*.

### Linking model

To link cortical responses to the perception of motion visibility, we modeled the decision process of an observer as a comparison of linearly weighted responses from retinotopically defined visual cortical areas subject to additive Gaussian noise. The model assumed the form of a probit regression in which the difference in weighted cortical responses for the two stimuli were passed through a cumulative normal distribution to make a trial-by-trial prediction of a choice for the stimulus on the right (Fig. 4). The response to each visual stimulus for each cortical visual area was calculated from the parametric forms of population response functions for contrast and coherence, as defined above. When validating the model assumptions such as additive noise and lack of choice history terms, we used the parameters for the population response functions that were fit to passive viewing data^48^. To test whether fixed or flexible readouts were needed to explain task performance, we used parameters for the population response functions fit to BOLD data collected during task performance, as described above. The linking model parameters that were fit by maximum likelihood estimation to the behavioral data were the weights for each visual area (in different versions of the model, we either fit all eight visual areas or subsets of visual areas) and a bias term to account for any propensity to choose one side over the other. For the fixed readout, there was one set of cortical weights for both tasks, and for the flexible readout, there were two sets of weights, one for each task. We describe in more detail the specifics of the model below.

We used the population response functions (Eqs. 4 and 5) to simulate the trial-by-trial response of visual cortical areas to stimuli in the contralateral hemifield (Eq. 6). The parameters of the functions were either from the fit to passive viewing data or during task performance. Summing the response for contrast and coherence assumes that the responses to contrast and coherence are independent of each other, which we showed to be the case in Birman and Gardner^48^.

To obtain the “readout” of this representation from multiple cortical areas, we proceeded by linearly weighting the area responses (Fig. 4). The full readout with all visual areas was computed with the following equation:

7 $$R_{{\mathrm{patch}}}(s_{{\mathrm{con}}},s_{{\mathrm{coh}}}) \!= \beta _{{\mathrm{V}}1}R_{{\mathrm{V}}1}(s_{{\mathrm{con}}},s_{{\mathrm{coh}}}) + \beta _{{\mathrm{V}}2}R_{{\mathrm{V}}2}(s_{{\mathrm{con}}},s_{{\mathrm{coh}}}) + ... + \beta _{{\mathrm{MT}}}R_{{\mathrm{MT}}}(s_{{\mathrm{con}}},s_{{\mathrm{coh}}})$$

where the response for each area on the right side of the equation is computed according to Eq. 6. Each *β* was a free parameter, which set the weight assigned to cortical areas in the readout process. We use the phrase fixed readout to refer to a model in which there are eight cortical readout weights in total (one for each cortical area) shared across the two task conditions. Implicitly the *fixed readout* model therefore assumes that the measured cortical responses must differ between task conditions to accommodate changes in behavior. We use the phrase *flexible readout* when 16 weights were allowed, i.e., a separate weight for each task for each cortical area. In addition to the eight cortical area models, we also fit models in which we only used the response of areas V1 and MT, the most contrast and coherence sensitive human cortical areas, respectively^48^.

To compute the probability of an observer choosing the stimulus on the right, we passed the difference in response to the two stimuli through a cumulative normal distribution^51^:

8 $$P_{{\mathrm{right}}}\left( {s_{{\mathrm{con,left}}},s_{{\mathrm{con,right}}},s_{{\mathrm{coh,left}}},s_{{\mathrm{coh,right}}}} \right) = \,\, {\it{\Phi} }(R_{{\mathrm{right}}}(s_{{\mathrm{con,right}}},s_{{\mathrm{coh,right}}}) \\ - R_{{\mathrm{left}}}(s_{{\mathrm{con,left}}},s_{{\mathrm{coh,left}}}) + \beta _{{\mathrm{bias}}})$$

where *R*_right_ and *R*_left_ are the weighted cortical responses to the two stimuli on each trial, as calculated using Eq. 7. *β*_bias_ accounts for bias to one side and *Φ* is the cumulative probability of a normal distribution with *μ* = 0 and *σ* = 1.

In the linking model, we allowed an additional parameter *λ* to capture the observer's lapse rate, modifying Eq. 8:

9 $$P_{{\mathrm{right}}}\left( {s \ldots ,\lambda } \right) = \frac{\lambda }{2} + (1 - \lambda ){\it{\Phi }}(R_{{\mathrm{right}}}(s \ldots ) - R_{{\mathrm{left}}}(s \ldots ) + \beta _{{\mathrm{bias}}})$$

We empirically estimated the lapse rate by finding the rate of observer errors on trials with a stimulus strength far above threshold. Because we occasionally reset the step size in the staircases, we were able to record a non-negligible number of trials with large stimulus increments, from these we selected trials in which the increment was at least 15% for contrast or 40% for coherence, which corresponded to increments of at least 2× threshold (15% and 40% also correspond to the maximum increment, which could be shown at the highest base strength of contrast and coherence, respectively). Computed in this way, *λ* varied from 0% to 7% (mean 3.0%, 95% CI [1.94, 4.56]).

We fit all variants of the linking model with maximum likelihood estimation using the active-set algorithm as implemented by the function *fmincon* in MATLAB. To avoid getting trapped in local minima, we randomized the starting parameters and repeated the fitting procedure multiple times.

We fit the linking model both within observers and across observers to test for generalization. Several observers were involved in both the experiments reported here as well those reported in Birman and Gardner^48^ and so their linking models could be fit within observer. To ensure generalization, we also computed the average population response functions and used those to fit the linking model to the individual perceptual measurements from each of the 21 observers, including those who did not have within-subject measurements of cortical responses. For the population response functions estimated from passive viewing data, the averaged-physiology and within-subject models had similar cross-validated log-likelihoods, $\log \left( {\frac{{{\cal{L}}_{{\mathrm{average}}}}}{{{\cal{L}}_{{\mathrm{within}}}}}} \right) = - 2.22$, 95% CI [−8.18, 4.71]. This suggests that the population response functions were similar across subjects and that noise in the physiological measurements is reduced by averaging across observers. For the measurements during task performance, there was a large improvement from using averaged-physiology data, $\log \left( {\frac{{{\cal{L}}_{{\mathrm{average}}}}}{{{\cal{L}}_{{\mathrm{within}}}}}} \right) = 34.6$, 95% CI [−6.4, 185.4], presumably due to the lower signal-to-noise ratio in those data because the stimulus was limited to 0.5 s.

### Linking model variants

To capture bias due to past choices^54,55^, we tested models with additional stay/switch bias parameters. We added four additional parameters—two that absorbed bias after correct responses (usually found to be a bias toward the same side) and two that absorbed bias after incorrect responses (usually found to be switching after errors). For clarity, we show Eq. 8 modified, but this model was still fit with the lapse rate (Eq. 9):

10 $$P_{{\mathrm{right}}}(s \ldots ) =\,\, {\it{\Phi }}(R_{{\mathrm{right}}}\left( {s_{{\mathrm{con}}},s_{{\mathrm{coh}}}} \right) - R_{{\mathrm{left}}}\left( {s_{{\mathrm{con}}},s_{{\mathrm{coh}}}} \right) + \beta _{{\mathrm{bias}}} + \beta _{{\mathrm{left,correct}}}C_{{\mathrm{left}}} \\ + \beta _{{\mathrm{right,correct}}}C_{{\mathrm{right}}} + \beta _{{\mathrm{left,incorrect}}}I_{{\mathrm{left}}} + \beta _{{\mathrm{right,incorrect}}}I_{{\mathrm{right}}})$$

where *C* and *I* are binary variables set by whether the last trial was correct or incorrect, respectively, and had a response on the corresponding side (i.e., *C*_left_ = 1 if the observer chose left on the last trial and was correct).

We also fit an efficient selection variant of the linking model where responses are weighted according to their magnitude during active viewing^3,28^. In this version of the model, the responses in each cortical area were raised to an exponent *ρ*, multiplied by the cortical readout weights, and then the exponent root was taken before passing through the cumulative normal. The effect of this transformation is that an area which has a larger base response, through the exponential, will dominate the final signal. Again, for clarity we show this modification for Eq. 8 but the full model included lapse rates (Eq. 9):

11 $$P_{{\mathrm{right}}}(s \ldots ) = {\it{\Phi }}\left(\root {\rho } \of {{R_{{\mathrm{right}}}(s_{{\mathrm{con}}},s_{{\mathrm{coh}}})^\rho - R_{{\mathrm{left}}}(s_{{\mathrm{con}}},s_{{\mathrm{coh}}})^\rho + \beta _{{\mathrm{bias}}}}}\right)$$

The linking model described so far makes the assumption that sensory noise limiting perception is additive, i.e., independent of stimulus strength, but we also tested a variation with noise that increased with sensory response strength. If readout was limited by the variability of individual or small groups of correlated neurons, we might expect sensitivity to be subject to noise, which increases with response. We tested this Poisson variant of the model by setting the variance of the noise (i.e., *σ*^2^ in Eq. 8) to the average population response in the two dot patches, prior to being passed through the readout weights. Following the equations above, this computation is done by averaging the response across areas for each dot patch:

12 $$\sigma _{{\mathrm{patch}}}^2 = \frac{{R_{{\mathrm{V}}1} + R_{{\mathrm{V}}2} \ldots + R_{{\mathrm{MT}}}}}{N}$$

where *N* is the number of areas averaged and *R*_area_ is computed using Eq. 6. We based the noise on the signal prior to readout under the assumption that Poisson noise would be generated by spiking variability occurring in the sensory system.

### Interpreting linking model parameters

Using the fit model parameters, we were able to determine an estimate of the magnitude of noise limiting an observer’s perceptual sensitivity in units of BOLD percent signal change. Because we set *σ* = 1 in the cumulative normal function of Eq. 7, we can estimate the noise in the sensory representation from the weight parameters. According to Eq. 8, a unit input difference between *R*_right_ and *R*_left_ will allow the observer to achieve threshold performance. It follows then that the *β* weights (Eq. 7) can be interpreted as scaling the raw BOLD responses such that a unit difference in weighted response gives rise to threshold performance. Assuming a standard signal-detection model where perceptual sensitivity (*d’*) is equal to the difference in responses divided by the standard deviation of the noise, a small *β* weight would suggest a large amount of noise is limiting perception as it would take a very large difference in response to get threshold performance. Conversely, a large *β* weight would suggest the opposite, that only small differences in response are needed for threshold performance. More formally, if one considers just one area, such as V1:

13 $${\mathrm{Threshold}}\,{\mathrm{performance}}\,\left( {d\prime = 1} \right) = \frac{{\left( {R_{{\mathrm{V}}1,{\mathrm{right}}} - R_{{\mathrm{V}}1,{\mathrm{left}}}} \right)}}{{\sigma _{{\mathrm{V}}1}}} = \beta _{{\mathrm{V}}1}( {R_{{\mathrm{V}}1,{\mathrm{right}}} - R_{{\mathrm{V}}1,{\mathrm{left}}}} )$$

Therefore, the *β* weights are inversely proportional to the implied neural noise, *σ*, of the representation, which limits perception.

To recover the model’s JNDs (Fig. 2), we proceeded analytically. As described above, because we fit the additive noise model with the noise parameter *σ* = 1 the population response functions, after scaling by the beta weights, are in units of standard deviations. To find the JND relative to a base stimulus strength, we calculated the increment in signal needed to increase the readout response by one, equivalent to *d’* = 1. This is because when *σ* = 1 we can reduce

14 $$d\prime = 1 = \frac{{R({\mathrm{base}} + {\mathrm{increment}}) - R({\mathrm{base}})}}{\sigma }$$

to simply

15 $$R({\mathrm{base}} + {\mathrm{increment}}) - R({\mathrm{base}}) = 1.$$

### Model comparison

To compare the different variants of the linking model, we used the cross-validated log-likelihood ratio and Tjur’s coefficient of discrimination^52^. Each variation of the linking model was fit in a ten-fold cross-validation procedure. In all, 10% of the data was reserved for validation while the remaining 90% was used to train. The log-likelihood was computed for each validation set and summed across all ten-folds. To compare any two variations of the linking model, we computed their likelihood ratio (i.e., the difference in total log-likelihood). The cross-validated log-likelihood ratio is similar in principle to measures of information criterion and sometimes referred to as the cross-validated information criterion. When the difference in this statistic between two models is large, e.g., >10, it indicates a substantial improvement in model fit. We use the cross-validated log-likelihood ratio rather than other information criterions (e.g., Akaike information criterion or Bayesian information criterion) because the cross-validation procedure already penalizes models with additional parameters for over-fitting. Although the cross-validated log-likelihood is useful for model comparison, it is difficult to interpret its absolute magnitude in isolation. To help with interpretation, we also report the cross-validated coefficient of discrimination CD.

16 $${\mathrm{CD}} = \mu _{{\mathrm{right}}} - \mu _{{\mathrm{left}}}$$

where *μ*_right_ is the model’s average predicted likelihood (Eq. 9) of a rightward choice for validation trials when the observer chose right and *μ*_left_ when the observer chose left. If the model predicts choices perfectly, then *μ*_right_ would be 1 and *μ*_left_ would be 0, giving a value for CD of 1. If the model is at chance at predicting choices, then CD would be 0. CD therefore indexes the difference between the centers of the trial-by-trial prediction distributions, and although not a true measure of variance explained, it shares many of the properties of *r*^2^ and is interpretable in a similar manner^52^.

### Reporting summary

Further information on research design is available in the Nature Research Reporting Summary linked to this article.

## Supplementary information

Supplementary Information

Reporting Summary

## Data Availability

The BOLD imaging and behavioral data that support the findings of this study are available in the Open Science Framework with the identifier 10.17605/OSF.IO/J6TMA.

## Code availability

Code to reproduce the main findings is included with the data. The full code to reproduce all figures and results are available on Github with the identifier 10.5281/zenodo.2805507.
