# Supplementary material for: A flexible readout mechanism of human sensory representations
Source: Nat Commun. 2019 Aug 2;10:3500. doi: 10.1038/s41467-019-11448-7 (PMC6677769; doi:10.1038/s41467-019-11448-7)
Supplement: Supplementary file 1 — Supplementary Information [file 41467_2019_11448_MOESM1_ESM.pdf]

Supplementary Note 1 related to Figure 7

Observers who we measured physiology for (black bars, Fig. 6c and d) show a larger improvement in model fit compared to the other observers (gray bars). One explanation for this effect is that the observers we measured in the scanner were better able to ignore the irrelevant feature due to having more practice. The fixed readout model, which predicts an inability to ignore the irrelevant feature, would then fail more dramatically for better-trained observers. Indeed, observers who were a part of the scanning were slightly better (n=11 observers, mean just-noticeable difference for contrast 5.62%, 95% CI [4.98, 6.50], and coherence 18.06%, 95% CI [16.32, 21.15]) compared to observers who did not participate in scanning (n=10, mean just-noticeable difference for contrast 10.02, 95% CI [6.23, 19.95], and coherence 21.40%, 95% CI [18.64, 25.03]).

Supplementary Note 2

An increase in additive offset during one task condition or the other could be used by an efficient selection model that weighs signals by their magnitude[1,2], e.g. selecting out V1 during the contrast task and MT during the coherence task. On average response magnitudes did increase a moderate amount when observers performed the task compared to the passive viewing condition, but these additive offsets were similar for both tasks (Fig. 5c). We found that the flexible model was a far better explanation than an efficient selection model (see Methods for implementation details), $\log\left(\frac{\mathcal{L}_{Flexible}}{\mathcal{L}_{Selection}}\right)$ = 130.39, 95% CI [109.66, 151.31], difference in CD, 0.30, 95% CI [0.28, 0.32].

Supplementary Note 3 related to Figure 8

Because observers were told at the start of each block (~65 trials or 4 minutes) whether or not catch trials would occur there is a concern that they could have split their attention, but we found no evidence for this. In other dual task settings there is a significant cost associated with performing two tasks at the same time[3], especially when one or both

tasks are difficult (near perceptual threshold). Note that we designed the catch trials to minimize this effect by making them rare and not providing feedback. If observers split their attention, we would expect to detect an increased just-noticeable difference (JND) on the cued main task. Instead, we found that the just-noticeable differences were similar: on runs with catch trials the contrast task JND increased by only 0.19% contrast, 95% CI [-0.19, 0.78], and for the coherence task by 0.74% coherence, 95% CI [-0.76%, 3.13%].

Supplementary references

1. Hara, Y. & Gardner, J. L. Encoding of graded changes in spatial specificity of prior cues in human visual cortex. *J Neurophysiol* **112**, 2834–2849 (2014).
2. Pestilli, F., Carrasco, M., Heeger, D. J. & Gardner, J. L. Attentional enhancement via selection and pooling of early sensory responses in human visual cortex. *Neuron* **72**, 832–846 (2011).
3. Sperling, G. & Melchner, M. The attention operating characteristic: examples from visual search. *Science* **202**, 315–318 (1978).
