# Supplementary material for: A flexible readout mechanism of human sensory representations
Source: Nat Commun. 2019 Aug 2;10:3500. doi: 10.1038/s41467-019-11448-7 (PMC6677769; doi:10.1038/s41467-019-11448-7)
Supplement: Supplementary file 2 — Reporting Summary [file 41467_2019_11448_MOESM2_ESM.pdf]

nature research

Corresponding author(s): Daniel Birman

Last updated by author(s): Jul 8, 2019

Reporting Summary

Nature Research wishes to improve the reproducibility of the work that we publish. This form provides structure for consistency and transparency in reporting. For further information on Nature Research policies, see Authors & Referees and the Editorial Policy Checklist.

Statistics

For all statistical analyses, confirm that the following items are present in the figure legend, table legend, main text, or Methods section.

n/a	Confirmed	
☐	☒	The exact sample size (n) for each experimental group/condition, given as a discrete number and unit of measurement
☒	☐	A statement on whether measurements were taken from distinct samples or whether the same sample was measured repeatedly
☐	☒	The statistical test(s) used AND whether they are one- or two-sided<br>*Only common tests should be described solely by name; describe more complex techniques in the Methods section.*
☒	☐	A description of all covariates tested
☒	☐	A description of any assumptions or corrections, such as tests of normality and adjustment for multiple comparisons
☐	☒	A full description of the statistical parameters including central tendency (e.g. means) or other basic estimates (e.g. regression coefficient) AND variation (e.g. standard deviation) or associated estimates of uncertainty (e.g. confidence intervals)
☐	☒	For null hypothesis testing, the test statistic (e.g. F, t, r) with confidence intervals, effect sizes, degrees of freedom and P value noted<br>*Give P values as exact values whenever suitable.*
☒	☐	For Bayesian analysis, information on the choice of priors and Markov chain Monte Carlo settings
☒	☐	For hierarchical and complex designs, identification of the appropriate level for tests and full reporting of outcomes
☐	☒	Estimates of effect sizes (e.g. Cohen's d, Pearson's r), indicating how they were calculated

Our web collection on statistics for biologists contains articles on many of the points above.

Software and code

Policy information about availability of computer code

Data collection	Data in this study was collected using MATLAB and MGL (doi:10.5281/zenodo.1299497).
Data analysis	Analysis was performed using MATLAB, MGL, and custom scripts. The full code to reproduce all figures and results are available on Github with the identifier 10.5281/zenodo.2805507.

For manuscripts utilizing custom algorithms or software that are central to the research but not yet described in published literature, software must be made available to editors/reviewers. We strongly encourage code deposition in a community repository (e.g. GitHub). See the Nature Research guidelines for submitting code & software for further information.

Data

Policy information about availability of data

All manuscripts must include a data availability statement. This statement should provide the following information, where applicable:

- Accession codes, unique identifiers, or web links for publicly available datasets
- A list of figures that have associated raw data
- A description of any restrictions on data availability

The BOLD imaging and behavioral data that support the findings of this study are available in the Open Science Framework with the identifier 10.17605/OSF.IO/J6TMA.

Field-specific reporting

Please select the one below that is the best fit for your research. If you are not sure, read the appropriate sections before making your selection.

☐ Life sciences ☒ Behavioural & social sciences ☐ Ecological, evolutionary & environmental sciences

For a reference copy of the document with all sections, see nature.com/documents/nr-reporting-summary-flat.pdf

Behavioural & social sciences study design

All studies must disclose on these points even when the disclosure is negative.

Study description	This study involves psychophysical and cortical measurements of human observers performing a selective visual attention task. The study was designed as a within-subject experiment: sufficient data was collected from each individual to validate the results, and aggregated results are presented.
Research sample	The research sample consisted of majority graduate students and undergraduate students at Stanford University. After exclusions, 13 participants were female, 8 male, and the age range was 18-55 years (mean age 28 y). The scanning sample was a subset of this group, 7 female, 3 male, age range 19-36 years (mean age 26 y).
Sampling strategy	Participants were drawn from a convenience sample at Stanford University and paid for their time.
Data collection	Participants performed the selective visual attention task in a dark room while eye tracking was performed. An experimenter sat in an adjacent room and monitored data quality measures which were agnostic to the study design. Each participant performed six one-hour sessions plus training, to collect 1167-3652 trials of data (mean 2467 trials). See below for details about scanning.
Timing	Data was collected from May 2016 to August 2016.
Data exclusions	Eight participants were excluded due to an inability to maintain fixation, either due to poor calibration quality or because they moved their eyes towards or away from the stimulus during the task.
Non-participation	No participants dropped out of the experiment.
Randomization	The within-subject design did not require randomization to separate groups.

Reporting for specific materials, systems and methods

We require information from authors about some types of materials, experimental systems and methods used in many studies. Here, indicate whether each material, system or method listed is relevant to your study. If you are not sure if a list item applies to your research, read the appropriate section before selecting a response.

Materials & experimental systems

n/a	Involved in the study	
☒	☐	Antibodies
☒	☐	Eukaryotic cell lines
☒	☐	Palaeontology
☒	☐	Animals and other organisms
☐	☒	Human research participants
☒	☐	Clinical data

Methods

n/a	Involved in the study	
☒	☐	ChIP-seq
☒	☐	Flow cytometry
☐	☒	MRI-based neuroimaging

Human research participants

Policy information about studies involving human research participants

Population characteristics	See above.
Recruitment	Participants were recruited through a website hosted by the Stanford Department of Psychology (stanfordpsychpaid.sona-systems.com).
Ethics oversight	Procedures were approved in advance by the Stanford Institutional Review Board on human participants research and all observers gave prior written informed consent.

Note that full information on the approval of the study protocol must also be provided in the manuscript.

Magnetic resonance imaging

Experimental design

Design type	An event-related design was used.
Design specifications	Each participant first performed a one-hour anatomy and retinotopy session. They then completed two 90-minute sessions each consisting of eight 7-minute runs. Within each run participants were exposed to sixteen combinations of contrast and motion coherence base strengths while either performing a task on the contrast stimulus or the coherence stimulus. In total there were 32 conditions (4 contrasts * 4 coherences * 2 tasks). We recorded approximately twenty trials of each condition, for each participant.
Behavioral performance measures	Participant's perceptual sensitivity (d') was measured to evaluate their performance on the task during scanning runs.

Acquisition

Imaging type(s)	functional
Field strength	3T
Sequence & imaging parameters	Functional images were obtained using a whole-brain T2*-weighted two-dimensional gradient-echo acquisition (FOV = 220mm, TR = 500 ms, TE = 30 ms, flip angle = 46 deg, 7 slices at multiplex 8 = 56 total slices, 2.5 mm isotropic). In addition, two whole-brain high-resolution T1-weighted 3D BRAVO sequences were acquired (FOV=240mm, flip angle=12 deg, 0.9 mm isotropic) and averaged to form a canonical anatomical image which was used for segmentation, surface reconstruction, session-to-session alignment, and projection of data onto a flattened cortical surface.
Area of acquisition	whole brain

Diffusion MRI ☐ Used ☒ Not used

Preprocessing

Preprocessing software	Preprocessing was performed using mrTools (doi:10.5281/zenodo.1299483) and included linear trend removal, high pass filtering (cutoff of 0.01Hz), and motion correction with a rigid body alignment using standard procedures.
Normalization	Data were not normalized.
Normalization template	n/a
Noise and artifact removal	n/a
Volume censoring	n/a

Statistical modeling & inference

Model type and settings	A finite impulse response model was used to deconvolve the responses to each stimulus condition.
Effect(s) tested	The magnitude of response was measured for each of the sixteen combinations of contrast and coherence base strengths in each of the two tasks.

Specify type of analysis: ☐ Whole brain ☒ ROI-based ☐ Both

Anatomical location(s)	Visual cortical areas V1-V4, V3A/B, V7 (IPS0), and MT (hMT+) were identified using the population receptive field method and standard criteria.
Statistic type for inference (See Eklund et al. 2016)	n/a
Correction	n/a

Models & analysis

n/a	Involved in the study
☒	☐ Functional and/or effective connectivity
☒	☐ Graph analysis
☒	☐ Multivariate modeling or predictive analysis
